# Clinical features, Outcomes and Molecular Profiles of Drug Resistance in Tuberculous Meningitis in non-HIV Patients

**DOI:** 10.1038/srep19072

**Published:** 2016-01-07

**Authors:** Jingya Zhang, Xuejiao Hu, Xin Hu, Yuanxin Ye, Mengqiao Shang, Yunfei An, Haimei Gou, Zhenzhen Zhao, Wu Peng, Xingbo Song, Yanhong Zhou, Mei Kang, Yi Xie, Xuerong Chen, Xiaojun Lu, Binwu Ying, Lanlan Wang

**Affiliations:** 1 Department of Laboratory Medicine, West China Hospital, Sichuan University, Chengdu 610041, Sichuan Province, P.R China; 2 Department of Neurosurgery, West China Hospital, Sichuan University, Chengdu 610041, Sichuan Province, P.R China; 3 Division of Pulmonary Disease, Department of Respiratory Medicine, West China Hospital, Sichuan University, Chengdu 610041, Sichuan Province, P.R China

## Abstract

Tuberculous meningitis continues to be a serious problem for physicians because it is difficult to make an early diagnosis and the consequences of delaying treatment are severe. The objective of this study is to provide data for the optimization of diagnostic and timely treatment of tuberculous meningitis. Of the 401 human immunodeficiency virus (HIV)-negative tuberculous meningitis patients in our study, 332 were found to have an impaired blood brain barrier (82.8%). Nearly 17.0% of patients failed to be timely diagnosed. Headache (53.6%) and fever (48.6%) were the most common features, and Computed Tomography/Magnetic Resonance Imaging (CT/MRI) detected 96 patients (23.9%) with abnormal meningeal imaging. Cerebrospinal fluid real-time polymerase chain reaction was positive in 73.8% of the tuberculous meningitis patients, whereas, smears and cultures detected only 6.7% and 5.2%, respectively. Further analysis identified striking differences between drug-resistant and drug-susceptible tuberculous meningitis. Patients with drug resistance correlated with grave prognosis. Tuberculous meningitis diagnosis should overall embody clinical symptoms, laboratory and cerebral imaging findings, and more sensitive diagnostic approaches are still warranted. Our data suggest cerebrospinal fluid polymerase chain reaction for mycobacterial DNA and molecular drug susceptibility testing as routine assays for suspected tuberculous meningitis patients, and observation of the blood brain barrier function could be performed for individual management.

Tuberculous meningitis (TBM) is the most severe form of tuberculosis (TB) and causes death or severe neurological defects in more than half of affected patients, despite advancements in available anti-tuberculosis treatments^1^,^2^. Early identification of TBM is crucial for treatment success^3^.The presence of concomitant diseases in TBM patients contributes to diversity in the patients’ clinical manifestations and the results of laboratory examinations of cerebrospinal fluid (CSF) and cerebral Computed Tomography/Magnetic Resonance Imaging (CT/MRI). The diagnosis of TBM remains problematic despite many significant advances in diagnostic techniques, and treatment has become more challenging because of the emergence of Human Immunodeficiency Virus (HIV) and drug-resistant strains of *Mycobacterium tuberculosis* (MTB)^2^,^4^. TBM continues to be a serious problem for physicians because it is difficult to make an early diagnosis and because the consequences of delaying treatment are severe^4^. In the absence of a specific objective diagnostic method, the diagnosis of TBM is currently based mainly on the clinician’s experience, non-specific symptoms, and a laboratory examination^5^,^6^,^7^,^8^,^9^,^10^,^11^,^12^. Therefore, understanding the characteristics of TBM is very important for the diagnosis of this disease. Recently, a number of studies have focused on describing the etiology, clinical presentation, and outcomes of tuberculous meningitis in HIV-TB co-infected TBM and TBM in children^13^,^14^. However, studies on HIV-negative TBM patients, which represent a special group of TBM patients, have been neglected, and the data have been deficient for years. Here, we present 5 years of data in non-HIV infected patients diagnosed with TBM in southwest China. Our aim is to describe the clinical features, outcomes and molecular profiles of drug resistance characteristics in this special group.

## Results

### Clinical features, laboratory test results and prognoses in HIV-negative TBM patients in China

A total of 218 men and 183 women who were HIV-negative TBM patients were recruited. The age of the patients ranged from 11 to 84 years old, with a median age of 39 years old. Of these, 105 (26.2%) patients had a history of tuberculosis infection. In a clinical examination, 53.6% of the patients complained mainly of headache, whereas 48.6% of the patients complained mainly of fever. Physical examinations revealed that 24.9% of the patients exhibited signs of meningeal irritation, 26.2% had altered mentation, and 25.9% displayed confusion. The prevalence of all other symptoms and signs was less than 20%. However, these symptoms and signs of TBM patients were atypical and difficult to differentiate TBM from other neurological diseases.

Only 6.7% and 5.2% of the 401 patients had acid-fast positive and culture positive results in CSF samples, respectively. This rate was lower than rates reported in other studies^7^,^15^,^16^. The practice of collecting only a small volume of CSF in our hospital may partially explain these differences. According to the British Infection Association (BIA), the recommended volume of collected CSF for TBM patients is 15–17 ml and the recommended volume for culture and staining is 10 ml. The volume collected for CSF analysis in our hospital was only 3–4 ml. Real-time polymerase chain reaction (PCR) was more sensitive than conventional smear and culture for the diagnosis of TBM, with a positive rate of 73.8%, serving as an instant and effective diagnostic complementary tool for MTB detection in CSF. CT/MRI results detected 96 patients (23.9%) with meningeal enhancement, 69 patients (17.2%) with intracerebral tuberculoma and 47 patients (11.7%) with extracranial tuberculosis infection. In total, 21 patients died. The ages of these patients ranged from 12 to 64 years old, and 17 of these 21 patients exhibited CT/MRI evidence suggesting a diagnosis of TBM, whereas 14 of them had concomitant pulmonary tuberculosis. The clinical conditions in 298 of the patients (74.3%) improved, indicating that the therapeutic strategy used for TBM was effective (Table 1).

### Subgroup analysis results in HIV-negative TBM patients

According to the methods of Thwaites *et al.*^17^ and Marais *et al.*^18^, the patients were divided into three groups based on age (Table 2). Patients in group 1 were ≤15 years old, patients in group 2 were 15 to 36 years old, and patients in group 3 were ≥37 years old. There were 21 patients in group 1, 155 patients in group 2, and 225 patients in group 3. With respect to clinical symptoms, 43.1%, 40.4%, and 13.3% of the patients in group 3 complained of headache, fever, and vomiting, respectively. These percentages were lower than those in group 1 and group 2 (p < 0.05 for all). There were meningeal signs in 11.6% of the patients in group 3, which was lower than the 57.1% of patients with such signs in group 1 (p < 0.01) and 40% in group 2 (p < 0.01). The extra meningeal TB accounted for 18.1% of group 2, which was higher than the percentages in group 1 (0%, p < 0.05) and group 3 (8.4%, p < 0.01). A good recovery was observed in 85.8% of the patients in group 2, which was better than the 65.3% of patients who experienced a good recovery in group 3 (p < 0.01).

According to the guidelines proposed by Marais *et al.*^18^, we also classified 401 enrolled TBM patients according to their diagnostic scores calculated by initial examination results after admission. In group 1, 8 patients (38.1%) were considered to have definite TBM, 5 patients (23.8%) were considered to have probable TBM, 4 patients (19.0%) were considered to have possible TBM, and 4 patients (19.0%) were considered to have not TBM. In group 2, there were 52 (33.5%), 41 (26.5%), 37 (23.9%), and 25 (16.1%) patients in the definite, probable, possible, and not TBM groups, respectively. In group 3, there were 71 (31.6%), 61 (27.1%), 54 (24.0%), and 39 (17.3%) patients in the definite, probable, possible, and not TBM groups, respectively. Approximately 17.0% of the TBM patients might fail to be diagnosed in a timely manner and therefore would not receive appropriate therapy if clinicians establish the diagnosis of TBM based on only the symptoms and signs of meningitis, laboratory examinations of CSF and CT/MRI results. This may lead to a poor prognosis in these patients.

### Clinical features of 35 cases with drug susceptibility testing (DST)

Thirty-five patients (19 men and 16 women with a mean age of 42 years old) were randomly sampled from 401 TBM patients for MTBDRplus and MTBDRsl assays, and 17 (48.6%) of these patients had experienced a prior TB infection. As shown in Table 3, the most common clinical symptoms in these 35 patients were headache and fever (77.1%), followed by altered mentation (40%) and vomiting (31.4%). In the radiological assay (CT/MRI), meningeal enhancement (62.8%) and tuberculoma (42.9%) were the most common abnormalities. All 35 patients exhibited negative HIV-1 antibody test results. In all, 32 (91.44%) of the cases were followed up, and 27 (77.14%) of the cases exhibited a good recovery. All of these data were similar to the data for the full sample of 401 patients.

### Laboratory examination of CSF in HIV-negative TBM patients in China

According to the CSF analyses, an increased number of leukocytes, with a lymphocytic predominance of more than 50%, a decreased glucose concentration and a markedly increased protein concentration was detected in the CSF of most of the patients (Table 4). We did not calculate the CSF to plasma glucose ratio because the plasma was not collected simultaneously with the CSF. Of the 401 enrolled TBM patients analyzed by Protis software in our study, 332 were found to involve the BBB impairment.

In the 35 cases that underwent drug susceptibility testing (DST), the median CSF opening pressure was 170 cmH_2_O, and 14 (40.0%) had an increased CSF opening pressure (>200 cmH_2_O). The median CSF total leukocyte count was 140 × 10^6^/L, and 31 (88.6%) of the cases had an increased leukocyte count. The median CSF glucose level in these 35 cases was 2.1 mmol/L, and 26 (74.3%) of the cases had decreased glucose levels (<2.5 mmol/L). The median CSF protein concentration was 1.4 mg/dl, and 32 (91.4%) of the cases had an increased protein concentration (>0.45 mg/dl). The median Cl^−^ concentration in 35 of the cases was 111.4 mmol/l, and 28 (80.0%) of the cases had a decreased Cl^-^ concentration (<120 mmol/L) (Table 4).

In all, 23 of the 35 cases had a CSF IgG index and a CSF IgG synthesis rate analysis. Of these, 4 (17.4%) cases had a CSF IgG index that was increased, and 18 (78.3%) cases had a CSF IgG synthesis rate that was increased. Using Protis software, we found that 22 (62.9%) of the 35 cases exhibited BBB damage, 4 (11.4%) cases exhibited increased CSF IgG synthesis rates and BBB damage, and 1 case had only increased CSF IgG synthesis rates. The phenomenon of increased CSF IgG synthesis rates calculated by using Protis software is different from the routine CSF laboratory analysis results in that there was BBB damage in TBM. Results calculated by using Protis software may be concerned that IgG entering the CSF as a result of brain barrier damage can cause false positives in these cases.

### Drug-resistance of the 35 TBM with DST

As shown in Table 5, 14 (40.0%) of the cases exhibited resistance to one or more than one drug. Of these, 2 (5.7%) cases exhibited monoresistance to rifampicin (RFP), and 6 (17.1%) cases exhibited monoresistance to isoniazid (INH). A total of 4 cases (11.4%) were multi-drug resistant tuberculosis (MDR-TB), and another 2 cases exhibited resistance to more than one drug.

Further analysis of the results of genotypic assays indicated that among the analyzed mutations (Table 6), mutations at rpoB codons 518–525 accounted for 71.4% of the resistance to rifampicin, and mutations at katG315 accounted for 100% of the resistance to isoniazid. According to the MTBDRsl assay, the case resistant to fluoroquinolones had a genotype of GyrA at codons 92–97, and 2 cases of resistance to ethambutol had a genotype of embB at codon 306.

### Comparison between drug-resistant TBM and drug-sensitive TBM

As shown in Table 7, 13 (94.1%) of the 14 resistant cases exhibited extra-meningeal TB, whereas of the 21 sensitive cases, only one exhibited extra-meningeal TB. A subsequent analysis of clinical features indicated that 7 (50%) of the 14 resistant cases exhibited vomiting, whereas only 4 (19.0%) of the 21 sensitive cases reported vomiting (p < 0.05), which is consistent with high opening pressure. Overall, 4 (28.6%) of the 14 resistant cases remained disabled, whereas only one of the 21 sensitive cases remained disabled (p < 0.01).

Eleven of the 14 drug-resistant TBM patients (78.6%) exhibited high opening pressure, whereas only three of the 21 drug-sensitive patients (14.3%) exhibited high opening pressure (p < 0.01). No significant difference was observed in other comparisons with respect to the laboratory results of CSF.

As an anti-drug therapy, all patients were administered Isoniazid + Rifampicin + Pyrazinamide + Ethambutol + Levofloxacin (HRZEO) chemotherapy regimens as an initial treatment. Two cases exhibited a poor response to treatment and had their therapeutic regimen changed. Both of these cases exhibited resistance to both RFP and INH. Drug-sensitive TBM patients did not have their therapeutic regimen changed. Only two patients among the drug-resistant TBM patients had their therapeutic regimen changed.

### Comparison of clinical features, results of laboratory tests and prognoses between HIV-negative TBM patients and TBM patients in a high HIV prevalence setting

We compared our results to those of Marais *et al.*^19^ (Table 8), a study that focused on TBM in patients with a high HIV burden. There were significant differences in neurological symptoms, MRI/CT abnormalities, and outcomes between HIV-negative TBM patients and TBM patients in a high HIV prevalence setting (p < 0.05), with 25.9%, 19.5%, 6.0%, and 16.0% of the HIV-negative TBM patients exhibiting confusion, vomiting, seizures, and neck stiffness, respectively, and 53.5%, 33.3%, 13.2%, and 64.0% of the TBM patients in a high HIV prevalence setting exhibiting the same symptoms, respectively. The rate of MRI/CT abnormalities was found to be 4.5% for hydrocephalus in our results and 37.5% in those of Marais *et al.*^17^ (p < 0.01), and the rate of infarct was 3.2% in our results and 40.9% in Marais *et al.*^19^ (p < 0.01). The mortality was 5.2% in HIV-negative TBM patients and 37.5% in TBM patients in a high HIV prevalence setting (p < 0.01). These results indicated that clinical manifestations, prognoses, and imaging results are different between HIV-negative TBM patients and HIV-positive TBM patients.

## Discussion

From our statistical analysis of the clinical data as well as previous studies, we recognize that the diagnosis of TBM in children and adults is a major difficulty and may be obscured by days to weeks of non-specific meningitis symptoms in the early stage of the disease^20^,^21^ and the insensitive conventional CSF culture and smear methods^22^. According to the diagnostic criteria used to determine the diagnosis scores in Marais *et al.*^18^, which classifies cases as definite, probable, possible, or not TBM, more information may be obtained to support a diagnosis of TBM^18^. Unfortunately, approximately 17.0% of the confirmed TBM patients’ scores did not support a diagnosis of TBM immediately according to the guideline. As for non-culture tests such as CSF-PCR for mycobacterial DNA appears to contribute early diagnosis (78.3% PCR-positive in our study) of TBM. These results suggest us TBM should be considered in any patient presenting with meningitis symptoms in areas with a high incidence of TB, and clinical, microbiologic, and cerebral imaging findings should be combined appropriately to support a diagnosis of TBM. It’s worth noting that a positive CSF-PCR promotes the ability to rapidly diagnose infection with TBM. Implementing more rapid, economic and accurate diagnostic tests approaches may lead to an earlier diagnosis and improved therapeutic success, which is particularly important for a rapid-developing severe clinical condition as TBM.

Previous evidences have indicated that among anti-tuberculosis drugs, only lipophilic drugs such as isoniazid and pyrazinamide can penetrate the blood-brain barrier (BBB) and reach CSF concentrations that are active against MTB^23^.Whereas, 82.8% of the TBM patients were found to have an impaired BBB using Protis software analysis in our research. HRZEO chemotherapy regimen remained effective for most TBM cases, which suggest the effectiveness of anti-TB drugs such as rifampicin and ethambutol influenced by the permeability of the BBB and the leakage of these drugs from plasma to CSF through an increased BBB permeability. Adaptive mechanisms leading to BBB impairment and their influence on anti-therapy of TBM patients are needed to be investigated further. Our finding may provide new insight into determining more effective treatment strategies and optimal doses of the anti-tuberculosis drugs for TBM patients, especially for younger children, who need careful observation to achieve relative balance between the least toxicity and optimal therapeutic efficacy^24^. Future studies on monitoring a drug’s concentration in the CSF will help to modify sequential therapies.

Drug-resistance in TBM is the primary factor that influences tuberculosis treatment. In our previous study^25^, we found that the rate of MDR-TB was 32.14% in 28 cases of TBM. However, in the present study, 14 (40%) of 35 cases exhibited drug-resistance, and 4 (11.43%) out of 35 cases were found to be MDR-TB. We believe that the main reason for the difference in MDR rates between our experimental results is the small size of our samples, and the exact MDR-TB rate warrants further investigation in a larger random population sample. With respect to the phenotypic DST results, the S522Q mutation in the rpoB gene and the S315T mutation in the katG gene were the two most frequently observed mutations among multidrug-resistant and monoresistant strains. These results are different from our earlier report, which indicated that S531L in the rpoB gene was the most frequently observed mutation^25^. This difference may be the result of a difference in homology. In the MTBDRsl analysis, we observed that in fluoroquinolones (FLQ)-resistant samples, the D93G mutation in the GryA gene was the most frequently observed mutation, whereas for ethambutol (EMB), the M306V mutation in the embB gene was the only observed mutation. These results indicate the presence of either heterogeneous strains or mixed populations of MTB, which were all interpreted as resistant to the relevant drug. Further analysis revealed that drug-resistant TBM patients had a tendency to unfavorable response to anti-TB therapy and poor prognosis compared to drug-susceptible patients. Drug resistant tuberculosis, especially MDR-TB, was associated with long-term and complicated treatment, poor prognosis, and severe sequel^25^, and it also takes a huge toll on patients and medical workers^26^. Therefore, clinicians should consider the possibility of infection of drug resistant tuberculosis when TBM patients presented with heavier, recurrent condition, or the duration of delayed healing. It’s better to conduct DST immediately. And treatment methods should be adjusted accordingly.

The study of Marais *et al.*^19^ was conducted in a high HIV prevalence setting in which 88.3% of the enrolled TBM patients were co-infected with HIV. Significant differences were found with respect to neurological symptoms, MRI/CT abnormalities, and prognoses when we compared our results to those of Marais *et al.*^19^. Of note, adverse clinical outcomes were more common in HIV co-infected TBM patients. A large body of evidence has emerged suggesting that infection with the HIV plays an important role in the pathogenesis of TBM^14^,^19^,^27^. These compared results verify this point of view. Therefore, clinicians should consider the HIV status of a patient when diagnosing and treating TBM.

There are some limitations to this study. First, our study was based in southwest China and was focused on HIV-negative TBM, and our findings may not be applicable in other regions. Second, although 401 patients were included in this study, only 35 patients were involved in the genotype DST assay, and some significant differences between groups may not have been detected during the statistical analysis. It is necessary to accumulate more phenotypic and genotypic data for drug resistance in patients with TBM from different regions to obtain a better picture of drug resistance patterns in China. Third, data related to drug concentrations needs to be collected to more fully to describe the relationships between the effects of anti-TB treatments and the permeability of the BBB.

In conclusion, the present study was performed using samples from TBM patients without HIV infection. Our data suggest that TBM diagnosis should overall embody clinical symptoms, laboratory investigations and cerebral imaging findings. Among laboratory tests, molecular diagnostics achieves a rapid diagnosis and relatively higher sensitivity when compared to conventional smear and culture in CSF. Most TBM patients are accompanied with impaired BBB status. Patients with drug-resistance and HIV co-infection may easily evolve into unfavorable prognosis. We recommend that a molecular DST assay should be immediately requested if a positive CSF-PCR result is obtained and that the function of the BBB should be better analyzed for individual therapy requirement.

## Methods

### Patients

All details of the study design and all procedures involved were in accordance with the Declaration of Helsinki. Informed written consent was obtained from all enrolled patients. The protocols and procedures for the protection of human subjects were approved by the Ethics Committee of West China Hospital, Sichuan University (reference No. 198 (2014)). Furthermore, all of the methods were performed in accordance with the approved guidelines.

From January 2010 to September 2015, a total of 8164 cases of suspected meningitis in West China Hospital of Sichuan University underwent lumbar puncture (LP) and CSF analysis. Of these, 975 cases were suspected to have meningitis based on CSF abnormalities. A total of 401 cases (218 men and 183 women, median age = 39 years old) were finally diagnosed with TBM. Thirty-five patients (19 men and 16 women, median age = 42 years old) with CSF TB-DNA positive results were randomly sampled and submitted to drug susceptibility testing (DST).

### Procedures

All patients we enrolled underwent a clinical assessment and had blood drawn for hematological and biochemical tests. A lumbar puncture (LP) was performed, and CSF cell counts and protein and glucose concentrations were determined using routine CSF laboratory analysis techniques (Modular P800; Roche Diagnostics, Basel, Switzerland). CSF was centrifuged, and a portion of the deposit was examined using Gram, India ink, and Ziehl-Neelsen stains. The remaining portion of the sample was cultured on blood and chocolate agar for pyogenic bacteria and on Löwenstein–Jensen medium in liquid 7H9 media (Mycobacterium Growth Indicator Tubes, Becton Dickinson Microbiology Systems, Sparks, MD) for mycobacteria. Patients also underwent chest radiography and, in cases that were clinically indicated, a cranial computer tomography or magnetic resonance imaging scan was performed.

### Diagnostic method

A definite diagnosis was made by neurological physicians if the patients exhibited typical TBM clinical manifestations and/or cerebrospinal fluid pathological changes, including a positive result in the CSF in any one of the following three examinations: a direct acid fast bacilli (AFB) smear examination, culture by liquid or solid medium, or a nucleic acid amplification, such as PCR.

### CSF analysis

The cellular and biochemical analyses were performed using routine CSF laboratory analysis protocols (Modular P800; Roche Diagnostics, Basel, Switzerland).

### BBB function test

Protis software (Dade Behring Inc., USA), which could combine protein analytic results with Reibergrams histogram^28^,^29^ and make the analytic results more intuitive for users, was used to assess the synthesis of intrathecal immunoglobulin and the function of blood brain barrier. IgG index, which was calculated as (IgG_CSF_/IgG_Serum_)/(Alb_CSF_/Alb_Serum_) and applied to reflect the production rate of intrathecal immunoglobulin G (IgG), usually increases significantly in TBM^30^.

### HIV antibody detection

The presence of HIV antibodies in serum samples was determined using a Modular Analytics E170 automated immunoassay analyzer (HIV Combi; Roche Diagnostics).

### MDR-TB detection

For CSF that tested positive for TB DNA, the DNA was extracted using a NucliSens EasyMag (BioMerieux Lyon, French) and was then directly input into a Genotype MTBDRplus and MTBDRsl assay (BioMerieux Lyon, French).

### Statistical analysis

Clinical and laboratory features were compared. We applied a diagnostic scoring system that required the presence of symptoms or signs indicative of meningitis plus symptoms derived from clinical, CSF, or imaging criteria to calculate a patient’s score for each feature. Patients then moved up or down the diagnostic pyramid shown in Supplementary Figure S1 online. as subsequent results became available and were classified as definite, probable, possible, or not TBM according to diagnostic criteria^18^. The number of points required for a diagnosis of probable tuberculous meningitis (≥12 when imaging was available or ≥10 when imaging was not available) and the points awarded in each category were determined by reviewing studies that quantified the diagnostic contribution of specific variables^18^. Other statistical analysis was performed by SPSS17.0.

Ethical approval: Ethical approval was given by the Clinical Trials and Biomedical Ethics Committee of West China Hospital, Sichuan University (reference number: 198 (2014)).

## Additional Information

**How to cite this article**: Zhang, J. *et al.* Clinical features, Outcomes and Molecular Profiles of Drug Resistance in Tuberculous Meningitis in non-HIV Patients. *Sci. Rep.* **6**, 19072; doi: 10.1038/srep19072 (2016).

## Supplementary Material

Supplementary figure S1

## Figures and Tables

**Table 1:** Demographic profiles, clinical features and prognoses in non-HIV TBM patients.

Characteristics	Patients (n = 401)	
General information		
Age, median (IQR)	39	(23, 52)
Male, n (%)	218	54.4
Prior TB, n (%)	105	26.2
Clinical features at admission, n (%)		
Symptom		
Headache	215	53.6
Fever	195	48.6
Vomiting	78	19.5
Clinical sign		
Altered mentation	105	26.2
Seizures	26	6.0
Meningeal signs	100	24.9
Confusion	104	25.9
Coma	40	10.0
Any cranial nerve palsy	36	9.0
Hemiparesis or paraparesis	31	7.7
Laboratory results, n (%)		
Smear positive	27	6.7
MTB culture positive	21	5.2
PCR positive	296	73.8
MRI/CT abnormalities, n (%)		
Hydrocephalus	18	4.5
Meningeal enhancement	96	23.9
Infarct	13	3.2
Optochiasmatic arachnoiditis	9	2.2
Tuberculoma	69	17.2
Extrameningeal TB	47	11.7
Prognosis, n (%)		
Dead	21	5.2
Persistent vegetative state	3	0.7
Severe disability	37	9.2
Moderate disability	42	10.5
Good recovery	298	74.3

**Table 2:** Demographic profiles, clinical features and prognoses of the three age groups.

Characteristics	Group 1(n = 21)		Group 2 (n = 155)		Group 3 (n = 225)	
	Age ≤ 15		Age (16, 36)		Age ≥ 37	
General information						
Age, median (IQR)	14	(14, 15)	23	(19, 30)	50	(42, 61)
Male, n (%)	10	47.6	88	56.8	120	53.3
Prior TB, n (%)**	6	28.6	53	34.2	46	20.4
Clinical features at admission, n (%)						
Symptom						
Headache^Δ^**	15	71.4	103	66.5	97	43.1
Fever^Δ^**	14	66.7	90	58.1	91	40.4
Vomiting^ΔΔ^**	9	42.9	39	25.2	30	13.3
Clinical sign						
Altered mentation^Δ^	10	47.6	42	27.1	53	23.6
Seizures	2	1.0	10	6.5	14	6.2
Meningeal signs^ΔΔ^**	12	57.1	62	40.0	26	11.6
Confusion	6	28.6	43	27.7	55	24.4
Coma	4	19.0	17	11.0	19	8.4
Any cranial nerve palsy^Δ^*	4	19.0	19	12.3	13	5.8
Hydrocephalus	0	0	11	7.1	7	3.1
Meningeal enhancement	4	19.0	39	25.2	53	23.6
Infarct	0	0	5	3.2	8	3.6
Optochiasmatic Arachnoiditis	0	0	6	3.9	3	1.3
Tuberculoma	3	14.3	32	20.6	34	15.1
Extrameningeal TB^#^**	0	0	28	18.1	19	8.4
Prognosis, n (%)						
Dead	2	10.0	4	2.6	15	6.7
Persistent vegetative state	0	0	1	0.6	2	0.9
Severe disability*	0	0	8	5.2	29	12.9
Moderate disability*	1	4.8	9	5.8	32	14.2
Good recovery**	18	85.7	133	85.8	147	65.3
Grade by diagnostic scores, n (%)						
Definite	8	38.1	52	33.5	71	31.6
Probable	5	23.8	41	26.5	61	27.1
Possible	4	19.0	37	23.9	54	24.0
Not supported	4	19.0	25	16.1	39	17.3

*Group 2 vs. Group 3, p < 0.05; ^Δ^Group 1 vs. Group 3, p < 0.05; ^#^Group 1 vs. Group 2, p < 0.05.

**Group 2 vs. Group 3, p < 0.01; ^ΔΔ^Group 1 vs. Group 3, p < 0.01; ^##^Group 1 vs. Group 2, p < 0.01.

**Table 3:** Demographic profiles, clinical features and prognoses of the 35 patients with drug susceptibility testing.

Characteristics	TBM patients (n = 35)	
General information		
Age, median (IQR)	42	(24, 57)
Male, n (%)	19	54.2
Prior TB, n (%)	17	48.6
Clinical features at admission, n (%)		
Headache	27	77.1
Fever	27	77.1
Vomiting	11	31.4
Altered mentation	14	40.0
Focal deficit	5	14.3
Seizures	0	0
Meningeal signs	1	2.9
Coma	2	5.7
Hemiparesis or paraparesis	3	8.6
MRI/CT abnormalities, n (%)		
Hydrocephalus	1	2.9
Meningeal enhancement	22	62.8
Infarct	2	5.7
Optochiasmatic Arachnoiditis	0	0
Tuberculoma	15	42.9
Extrameningeal TB, n (%)	17	48.5
Prognosis, n (%)		
Dead	0	0
Persistent vegetative state	0	0

**Table 4:** Analysis of CSF in TBM patients.

CSF Analysis	Total TBM patients		TBM patients with DST	
	Median (IQR)	Abnormal (%)	Median (IQR)	Abnormal (%)
Opening pressure (cm H_2_O)	160 (105, 200)	94/401 (23.4)	170 (118, 194)	14/34 (40.0)
Total leukocyte count (x10^6^/l)	70 (10, 210)	305/401 (76.1)	140 (60, 310)	31/35 (88.6)
Lymphocytes (×10^6^/l)	14 (0, 110)	204/401 (50.9)	57 (0, 90)	19/35 (54.3)
CSF glucose (mmol/l)	2.31 (1.54, 3.37)	345/401 (86.0)	2.1 (1.72, 3.49)	26/35 (74.3)
CSF protein (mg/dl)	1.31(0.53, 2.2)	339/401 (84.5)	1.4 (0.03, 5.06)	32/35 (91.4)
CSF chlorinate (mmol/l)	117.1 (108.9,125.2)	263/401 (65.6)	111.4 (109.8, 121.3)	28/35 (80.0)
CSF IgG index	0.613 (0.51,0.735)	89/401 (22.2)	0.7 (0.464, 0.702)	4/23 (17.4)
CSF IgG synthesis rate	14.85 (0, 49.38)	329/401 (82.0)	13.1 (0, 127)	18/23 (78.3)

*p < 0.05.

**p < 0.01.

**Table 5:** Genotypes in drug susceptibility profiles of the GenoType MTBDRplus and MDRsl Assays.

Drug Resistance Patterns and Results of the MDRplus and MDRsl	Number with resistance	Percentage (%)
Resistance to any drug	14	40.0
Resistance to more than one drug	2	5.7
MDR-TB	4	11.4
Monoresistance to RFP	2	5.7
Monoresistance to INH	6	17.1
Monoresistance to FLQ	0	0
Monoresistance to KAN	0	0
Monoresistance to EMB	0	0
Resistance to REP + INH	3	8.6
Resistance to REP + EMB	1	2.9
Resistance to REP + INH + FLQ	1	2.9
Resistance to INH + FLQ + EMB	1	2.9

Notes: MDR-TB-multi-drug resistant tuberculosis; RFP-rifampicin; INH-isoniazid; FLQ-fluoroquinolones; KAN-kanamycin; EMB-ethambutol.

**Table 6:** Genotypes in MDRTBplus and MDRsl results to detect RIF, INH, FLQ, KAN and EMB resistance in 14 drug-resistant TB cases.

Drug Resistance	Resistance and GTplus & GTsl^a^ pattern			CSF Samples n (%)		
	Gene and Analyzed Codons	WT^b^	MUT^c^	Mono-drug resistance (n = 8)	MDR TB (n = 4)	Resistance to more than one drug (n = 2)
**Resistance to RIF**				**2 (28.6)**	**4 (57.1)**	**1 (14.3)**
	rpoB518-525	Δ6 (S522Q)		1 (14.3)	3 (42.9)	1 (14.3)
	rpoB530-533		Mut3 (S531L)	1 (14.3)	1 (14.3)	0
**Resistance to INH**				**6 (54.6)**	**4 (36.4)**	**1 (9.1)**
High level	katG 315		Mut1 (S315T1)	6 (54.6)	4 (36.4)	1 (9.1)
Low level	inhA			0	0	0
**Resistance to FLQ**				**0**	**1 (50.0)**	**1 (50.0)**
	GyrA92-97		Mut3C (D93G)	0	1 (50.0)	1 (50.0)
**Resistance to EMB**				**0**	**0**	**2 (100)**
	embB306		Mut1B (M306V)	0	0	2 (100)

^a^GTplus, the GenoType MTBDRplus Assay; GTsl, the GenoType MTBDRsl assay.

^b^WT, wild-type pattern with all respective bands; Δwt, omission of the respective wild-type band.

^c^MUT, mutations corresponding to the specific mutation probes in the tested gene.

**Table 7:** Clinical analysis of the drug resistant and non-resistant TBM patients.

Chemotherapy regimen changes (n = 2)	Drug-resistant patients (n = 14), n (%)	Non-resistant patients (n = 21), n (%)
	2 (100)	0
Clinical features		
Extrameningeal TB (n = 17)**	13 (94.1)	1 (4.7)
Headache (n = 27)	10 (71.4)	17 (80.9)
Fever (n = 27)	12 (85.7)	15 (71.4)
Altered mentation (n = 14)	6 (42.8)	8 (38.1)
Vomiting (n = 11)*	7 (50.0)	4 (19.1)
MRI/CT abnormalities		
Meningeal (n = 22)*	11 (78.57)	11 (52.4)
Tuberculoma (n = 15)	6 (42.9)	9 (42.9)
Prognosis		
Poor prognosis (n = 5)**	4 (28.6)	1 (4.7)
Chemotherapy for change (n = 2)*	2 (14.3)	0 (0)
CSF analysis		
High opening pressure (n = 14)**	11 (78.6)	3 (14.3)
High CSF leukocyte count (n = 31)	13 (92.9)	18 (85.7)
Low CSF glucose (n = 26)	9 (64.3)	17 (80.9)
High CSF protein (n = 32)	13 (92.9)	19 (90.4)
Low CSF chlorinate (n = 28)	12 (85.7)	16 (76.1)
High IgG index (n = 5)	2/9 (22.2)	3/14 (21.4)
CSF IgG synthesis rate (n = 23)	7/9 (77.8)	11/14 (78.6)

*p < 0.05.

**p < 0.01.

**Table 8:** Comparison of clinical features and laboratory results between HIV-negative TBM and HIV-positive TBM patients.

Characteristics	Patients			
	HIV-negative		HIV co-infection	
General information				
Male, n/N (%)	218/401	54.4	60/120	50.0
HIV status, n/N (%)**	0/401	0	106/120	88.3
Prior TB, n/N (%)	105/401	26.2	34/115	29.6
Neurological symptoms, n/N (%)				
Headache	215/401	53.6	61/114	53.5
Confusion**	104/401	25.9	61/114	53.5
Vomiting*	78/401	19.5	38/114	33.3
Seizures*	26/401	6.0	15/114	13.2
Neck stiffness**	64/401	16.0	73/114	64.0
Features of TB elsewhere, n/N (%)**	47/401	11.7	87/114	76.3
MRI/CT abnormalities, n/N (%)				
Hydrocephalus**	18/401	4.5	10/44	22.7
Meningeal enhancement	96/401	23.9	12/44	27.3
Infarct**	13/401	3.2	18/44	40.9
Outcomes, n/N (%)				
Dead**	21/401	5.2	45/120	37.5
Survived**	380/401	94.8	75/120	62.5
Diagnostic score, n/N (%)				
Definite	131/401	32.7	47/120	39.2
Probable	107/401	26.7	35/120	29.2
Possible	95/401	23.7	38/120	31.7

*p < 0.05.

**p < 0.01.
