# Supplementary material for: Clinical features, Outcomes and Molecular Profiles of Drug Resistance in Tuberculous Meningitis in non-HIV Patients
Source: Sci Rep. 2016 Jan 7;6:19072. doi: 10.1038/srep19072 (PMC4703954; doi:10.1038/srep19072)

Clinical features, Outcomes and Molecular Profiles of Drug Resistance in Tuberculous Meningitis in non-HIV Patients

Jingya Zhang[a,1], Xuejiao Hu[a,1], Xin Hu[a,2], Yuanxin Ye[1], Mengqiao Shang[1], Yunfei An[1], Haimei Gou[1], Zhenzhen Zhao[1], Wu Peng[1], Xingbo Song[1], Yanhong Zhou[1], Mei Kang[1], Yi Xie[1], Xuerong Chen[3], Xiaojun Lu[1,]*, Binwu Ying[1,]*, Lanlan Wang[1,]*

Supplementary Figure S1. Flowchart of the method used for the diagnosis of patients with tuberculous meningitis.

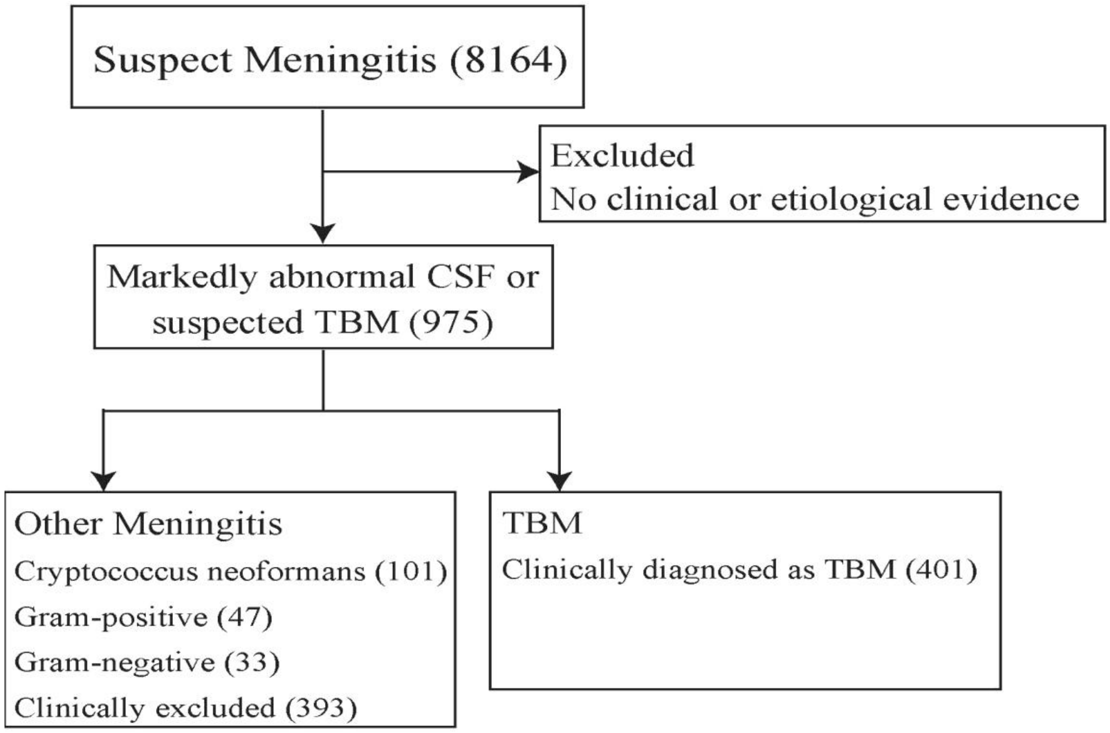

Supplement: Supplementary figure S1 [file srep19072-s1.pdf]
